# Adherence, belief, and knowledge about oral anticoagulants in patients with bioprosthetic heart valve replacement: a cross-sectional study

**DOI:** 10.3389/fphar.2023.1191006

**Published:** 2023-07-12

**Authors:** Yun-Xia Ni, Lu-Lu Liu, Huang Feng, Zhi Li, Chao-Yi Qin, Miao Chen

**Affiliations:** ^1^ Department of Cardiology, West China Hospital, Sichuan University/West China School of Nursing, Sichuan University, Chengdu, China; ^2^ Department of Cardiovascular Surgery, West China Hospital, Sichuan University, Chengdu, China; ^3^ Department of Cardiovascular Surgery, West China Hospital, Sichuan University/West China School of Nursing, Sichuan University, Chengdu, China

**Keywords:** bioprosthetic heart valve replacement, medication adherence, medication belief, knowledge, mediation effect, oral anticoagulation

## Abstract

**Aims:** To investigate adherence to oral anticoagulants among patients after mechanical heart valve (BHV) replacement and further examine the mediating role of medication belief in the relationship between knowledge and medication adherence.

**Background:** The number of patients who undergo BHV replacement has increased in recent years. Short-term anticoagulant therapy is recommended for patients after BHV replacement. However, little is known about adherence to oral anticoagulant therapy and the underlying mechanisms among patients with BHV replacement.

**Methods:** A cross-sectional study was conducted between September 2022 and November 2022. A convenience sample of 323 patients who underwent BHV replacement was recruited from a tertiary public hospital in Southwest China. Data were collected by using the 8-item Morisky Medication Adherence Scale, Beliefs about Medicines Questionnaire-specific, and the Knowledge of Anticoagulation Questionnaire. The mediation model was tested by Hayes’s PROCESS macro. The STROBE checklist was used.

**Results:** Approximately 17.3% of participants had low adherence, 47.1% had medium adherence, and only 35.6% reported high adherence to oral anticoagulants. Knowledge and necessity beliefs were positively related to medication adherence, while concern beliefs were negatively correlated with medication adherence. Medication belief mediated the relationship between knowledge and adherence to oral anticoagulants.

**Conclusion:** Patients with BHV replacement demonstrated relatively low adherence to oral anticoagulant therapy. Efforts to enhance medication adherence should consider improving patients’ knowledge and medication beliefs.

## 1 Introduction

The incidence of valvular heart disease (VHD) has increased in many countries (Iung et al., 2019; Cahill et al., 2021), affecting >2% of the population worldwide and resulting in rising mortality (Azari et al., 2020; Chen et al., 2020). Valve replacement is the only definitive treatment for people with severe VHD. Over 280,000 patients with VHD undergo heart valve replacements each year worldwide, and this number is expected to increase to 850,000 by 2050 (Bax Jeroen and Delgado, 2017). Two types of prosthetic heart valves can be implanted: mechanical heart valves (MHVs) and bioprosthetic heart valves (BHVs). MHVs have greater durability than BHVs but are more thrombogenic. Consequently, after MHV replacement, patients are required to receive life-long anticoagulant treatment and regular laboratory monitoring of the international normalized ratio (INR). In contrast, BHVs do not require lifelong anticoagulation and show excellent hemodynamic properties, which are similar to those of native valves (Kostyunin et al., 2020). The 2020 AHA/ACC guidelines (Otto et al., 2021) and 2021 ESC guidelines (Vahanian et al., 2022) for the management of valvular heart disease recommend BHV replacement in patients aged >65 years. With an aging population worldwide and the strong association between HVD and age (Rostagno, 2019), as well as economic growth, BHV replacement has increased in recent decades.

Despite obviating the lifelong necessity of anticoagulation for patients after BHV replacement, the risk of thromboembolism still exists in the immediate postoperative period, particularly during the first 3 months (Christersson et al., 2020; Carlin and Eikelboom, 2022). Accordingly, many national societies recommend oral anticoagulants for 3–6 months for patients with a BHV (Otto et al., 2021; CSfTaC Surgery, 2022; Vahanian et al., 2022). The benefits of short-term oral anticoagulation following BHV replacement have been reported by previous studies (Mérie et al., 2012; Ko et al., 2023), such as reducing the risk of embolism and improving long-term survival. The use of oral anticoagulants (e.g., warfarin) is complex and requires special care since the therapeutic window is narrow. Inappropriate use of oral anticoagulants can increase the risk of bleeding, thromboembolism, and even death (Otto et al., 2021). As such, it is crucial to maintain adequate adherence to anticoagulation therapy in patients after BHV replacement.

Medication adherence refers to the extent to which patients take their medications as prescribed by healthcare professionals (Osterberg and Blaschke, 2005). Despite the strong association between oral anticoagulant adherence and a lower incidence of adverse events, only 27.5%–50% of patients were found to be adherent to anticoagulants (Kim et al., 2011; Castellucci et al., 2015). Numerous studies have been undertaken to investigate anticoagulation adherence among patients who underwent MHV replacement (Park and Jang, 2021; Ni et al., 2022); however, anticoagulation adherence in patients with BHV replacement is far from being ignored. Although taking oral anticoagulants for patients after BHV replacement is only required in a short time (e.g., 3–6 months) (Vahanian et al., 2022), the narrow therapeutic window and potential serious complications make it necessary to fully understand adherence to oral anticoagulants for patients who undergo MHV replacement. To date, there is a dearth of evidence on the prevalence of adherence to anticoagulation therapy and factors associated with adherence among patients with BHVs.

Previous studies have found that oral anticoagulant adherence is affected by multiple factors, including sociodemographic characteristics (e.g., age, gender), knowledge, attitudes, perceptions of risks and benefits of anticoagulants, and family support (Castellucci et al., 2015; Park and Jang, 2021; Ni et al., 2022). In particular, the association between knowledge and adherence to oral anticoagulants has been directly examined in extensive studies (Davis et al., 2005; Smet et al., 2018); however, the results are inconsistent. For instance, Smet et al. (2018) found that a higher level of knowledge was significantly associated with higher adherence rates among patients taking oral anticoagulants. Conversely, a study conducted by Davis et al. (Davis et al., 2005) observed that knowledge does not necessarily warrant adherence to warfarin therapy. Nevertheless, past studies have demonstrated that medication adherence is enhanced by improving patients’ knowledge (Jiang et al., 2021). Among the patients who underwent BHV replacement, knowledge on the relationship between knowledge and medication adherence is lacking.

Research has found that medical belief, an individual’s perceptions of needs and concerns about medication, is one of the most important predictors of medication adherence (Taylor et al., 2020). For example, patients who believed that the benefits of medication outweighed its risks were more likely to take medications as prescribed. Instead, when the patients perceived more risks of taking medications, they tended to demonstrate nonadherence. However, studies on oral anticoagulants in the past have mainly focused on patients with long-term anticoagulant treatment (e.g., patients with MHVs) (Park and Jang, 2021), and whether the association between medication belief and adherence among patients with BHVs is similar to that among patients with MHVs remains unclear. Prior studies have shown that patients with lifelong oral anticoagulants were likely to recognize a burden on their daily life, such as regular laboratory monitoring, lifestyle modifications (e.g., alcohol consumption, diet and daily medication intake), side effects, and medical costs (Bartoli‐Abdou et al., 2018), which led to patients’ concerns about oral anticoagulants. Although patients after BHV replacement still needed laboratory monitoring and faced the risk of side effects when they took anticoagulants, the duration of oral anticoagulant intake was relatively short. Accordingly, medication belief in patients with BHVs was not necessarily consistent with that of patients with MHVs, which could eventually result in different adherence to oral anticoagulants.

**FIGURE 1:**
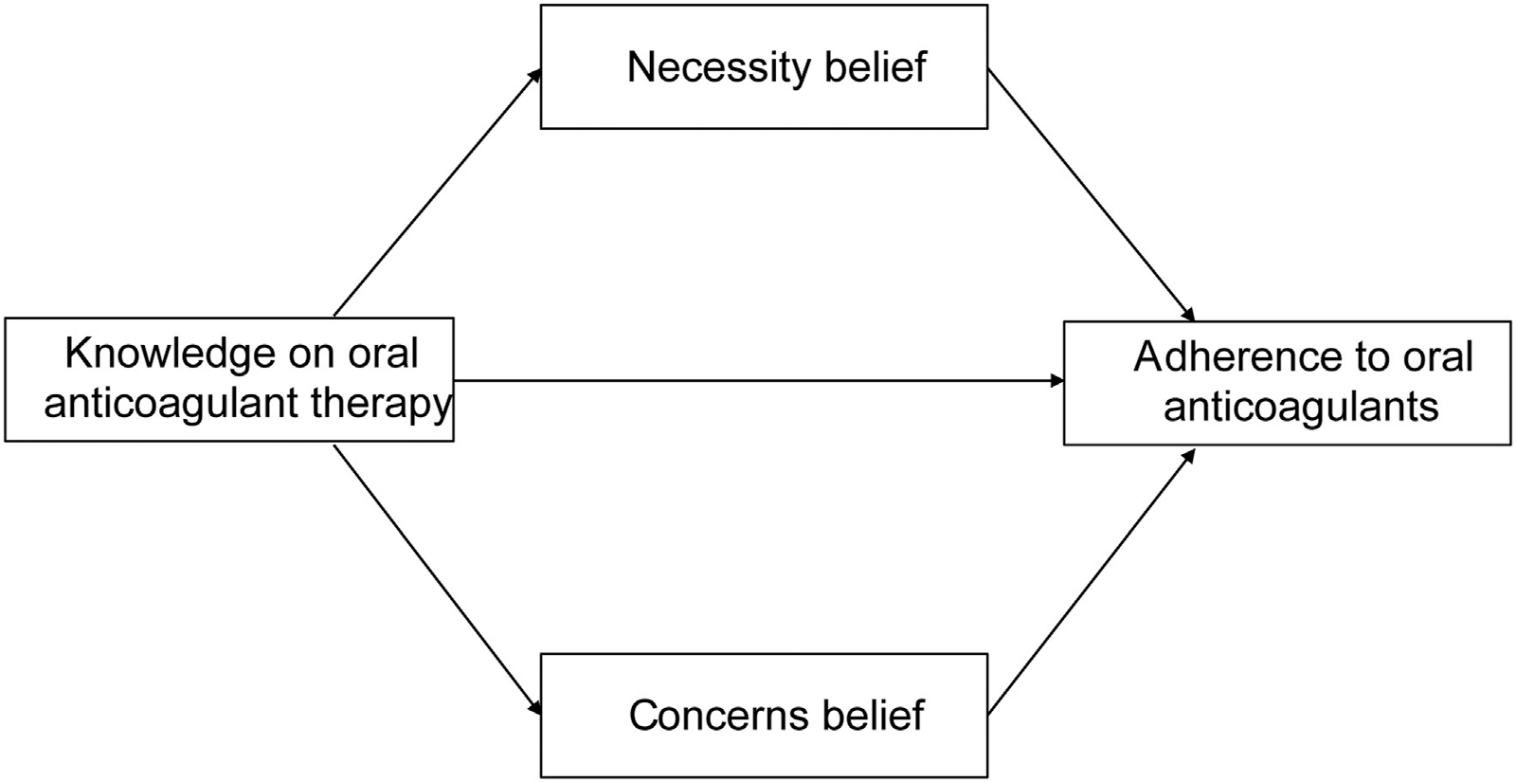
The hypothetical model.

Despite numerous studies exploring the association between knowledge, medication belief and medication adherence (Smet et al., 2018; Taylor et al., 2020), triangular relationship between these variables was less explored in patients who underwent BHV replacement. According to the knowledge, attitude, and practice (KAP) model (Dong et al., 2022), individuals’ knowledge is the basis for creating positive attitudes or beliefs, which in turn affect individuals’ behavior, such as medication intake behavior in our study. Therefore, we hypothesized that patients’ medication beliefs played a mediating role in the relationship between knowledge and adherence to oral anticoagulants among patients with BHV replacement (Figure 1).

The aim of the present study is to investigate adherence to oral anticoagulants among patients after BHV replacement and further examine the mediating role of medication belief in the relationship between knowledge and medication adherence.

## 2 Methods

### 2.1 Design, participants, and setting

A cross-sectional study was performed in a large-scale tertiary public hospital in Southwest China. The convenience sampling method was used to recruit participants who underwent bioprosthetic heart valve replacement in this hospital. The inclusion criteria were as follows: 1) aged over 18 years; 2) continued use of oral anticoagulants for at least 2 months after bioprosthetic heart valve replacement regardless of surgicalor transcathetertreatment; 3) ability to communicate; and d) voluntary participation in this study. Participants were excluded if 1) they had a diagnosis of dementia or cognitive impairment documented in their medical history or 2) they completely relied on caregivers for taking medicine.

To estimate the sample size, the number of participants required for mediation analysis with a bootstrapping procedure was calculated based on Fritz and MacKinnon’s suggestions (Fritz and Mackinnon, 2007). The sample size of 148 participants was estimated by considering a power of 0.80, a significance level of 5%, and a medium path coefficient of the indirect effect. We further estimated a 20% nonresponse rate, and ultimately, the sample size should be at least 185. Eligibility criteria were assessed by a cardiovascular nurse who had over 10 years of cardiovascular care experience. From the 403 patients who consecutively received bioprosthetic heart valve replacement between March 2021 and March 2022, a total of 323 patients who could be reached, interested in this study, and alive were recruited.

### 2.2 Measurement

#### 2.2.1 Adherence to oral anticoagulants

The Chinese version (Yan et al., 2014) of the 8-item Morisky Medication Adherence Scale (MMAS-8) (Morisky et al., 2008) was used to assess the patients’ adherence to oral anticoagulants. The MMAS–8 is a self-reported medication-taking behavior scale that consists of eight questions (Berlowitz et al., 2017). Questions 1–7 are measured with dichotomous response choices (yes or no) and the last item is measured using a 5-point Likert response. The total score of the MMAS‐8 ranges from 0 to 8, with a higher overall score indicating greater medication adherence. Respondents were categorized as low adherence (score < 6), medium adherence (6 ≤ score < 8), and high adherence (score = 8) (Bress et al., 2017). The Chinese version of the MMAS-8 shows good reliability and validity (Yan et al., 2014). The Cronbach’s α of this scale was 0.67 in the current study.

#### 2.2.2 Belief about oral anticoagulants

The Chinese version (Si, 2013) of the Beliefs about Medicines Questionnaire-specific (BMQ-specific) (Horne et al., 1999) was used to evaluate patients’ beliefs on the importance of oral anticoagulants and their concerns about their potential adverse effects. The BMQ-specific consists of 10 items that are divided into two dimensions: necessity (5 items) and concerns (5 items). Each item is scored on a 5-point Likert-type scale (1 = strongly disagree, 2 = disagree, 3 = uncertain, 4 = agree, and 5 = strongly agree). The score for each dimension ranges from 5 to 25, with higher scores indicating stronger beliefs in necessity or concern for oral anticoagulants. The overall score is calculated by subtracting the concern score from the necessity score, which ranges from −20 to 20. A larger positive score indicates a stronger positive belief, namely, the participants believe that the benefits of oral anticoagulants outweigh their costs. Instead, a larger negative value indicates that participants believe that the costs of oral anticoagulants outweigh their benefits. The Chinese version of the BMQ-specific had a reported Cronbach’s α of 0.698 and 0.784 (Cai et al., 2020). In this study, the Cronbach’s α for the necessity and concern dimensions were 0.79 and 0.75, respectively.

#### 2.2.3 Knowledge of oral anticoagulant therapy

The participants’ knowledge of oral anticoagulant therapy was measured by the Knowledge of Anticoagulation Questionnaire (KAQ) developed by Si (2013). As our sample only included patients after bioprosthetic heart valve replacement, a slight adaptation of the KAQ was used. This tool comprises 10 questions to evaluate four aspects of oral anticoagulant therapy: 1) the purpose of taking anticoagulants; 2) occurrence and management of side effects; 3) interactions with food and other medications; and 4) lifestyle. Each item was rated on a dichotomous scale (1 = yes and 0 = no). The total score of the KAQ ranges from 0 to 10, with a higher score indicating a higher level of medication knowledge. The content validity index of the KAQ was 0.93, and Cronbach’s α was 0.89 (Si, 2013). In the current study, Cronbach’s α was 0.74.

#### 2.2.4 Demographic and clinical characteristics

The researcher-designed questionnaire was used to assess participants’ demographic characteristics, including age, gender, marital status, education level, employment status, household, and residence. Clinical characteristics (e.g., comorbidities, months of oral anticoagulant consumption, operation name, type of operation) were obtained by reviewing the patient’s medical records.

### 2.3 Data collection

Data were collected between September 2022 and November 2022. The research team contacted the participants over the phone and explained the purpose of this study. After obtaining each participant’s informed consent, the participant was asked to answer the demographic and self-reported variables in the questionnaire. Data were collected via telephone interviews by three cardiovascular nurses (over 5 years of clinical nursing experience), and all participants’ responses were recorded on the questionnaire. These three cardiovascular nurses received training by the research team prior to data collection to ensure the standardization of study procedures. Any inducements and hints were avoided during the telephone interview process. Data collection took approximately 30 min for each participant. The researcher reviewed the electronic medical records and collected the clinical characteristics of the participants.

### 2.4 Data analysis

Analysis was conducted using SPSS (version 26.0) and Hayes' PROCESS (version 4.0). Descriptive statistics (i.e., frequency, percentage, means, and standard deviations) were used to describe the participant’s demographic and clinical characteristics, as well as outcome variables. Pearson correlation was performed to test the relationships between adherence to oral anticoagulants, beliefs about oral anticoagulants, and knowledge of oral anticoagulant therapy. To examine the mediating role of beliefs about oral anticoagulants on the relationship between knowledge of oral anticoagulant therapy and adherence to oral anticoagulants, Hayes' PROCESS macro (model 4) with a bootstrapping procedure was used. If the bias-corrected (BC) 95% confidence interval does not include “zero”, the mediation effects are significant. The participants’ demographic and clinical characteristics (i.e., age, residence, number of comorbidities, and type of operation) were included as covariates because these characteristics were associated with the adherence to oral anticoagulants.

Due to the self-reported data in our study, common method variance bias was examined by Harman’s single factor test (Podsakoff et al., 2003). The results showed that eight factors had eigenvalues higher than one, and the first factor explained 25.45% of the total variance, which indicated that common method bias was not a serious problem in the present study. Multicollinearity was assessed by calculating tolerance and the variance inflation factor. The results demonstrated that the tolerance value was more than 0.2, and the VIF value was smaller than 2.5, indicating that collinearity was not a threat in this study.

## 3 Results

### 3.1 Participant characteristics

A total of 323 patients were involved in the study. The mean age was 68.41 (SD = 6.31) years, ranging from 39 to 89 years. The majority of participants were married (84.2%), not working (76.2%), and living with family (91.6%). Approximately 68.7% of participants had an educational level of junior high school or below. Most participants (58.8%) who underwent transcatheterprosthetic heart valve replacement had comorbidities (86.7%). The demographic and clinical characteristics of the participants are presented in Table 1.

**TABLE 1:** Demographic and clinical characteristics of participants (n = 323).

Variable		Frequency	Percentage (%)
Age in years, mean (SD)		68.41 (6.31)	
Gender	Male	157	48.6
	Female	166	51.4
Marital status	Married	272	84.2
	Not married	51	15.8
Education level	Illiterate	47	14.6
	Primary school	96	29.7
	Junior high school	79	24.5
	Senior high school	51	15.8
	College degree or above	50	15.5
Employment status	Working	77	23.8
	Not working	246	76.2
Household	With family	296	91.6
	Live alone	26	8.0
	Nursing Home	1	0.3
Residence	City	158	48.9
	County/town	82	25.4
	village	83	25.7
Operation name	MVR	61	18.9
	AVR	225	69.7
	DVR (MVR + AVR)	34	10.5
	TVR	2	0.6
	MVR + AVR + TVR	1	0.3
Type of operation	Surgical	133	41.2
	Transcatheter	190	58.8
Comorbidities	Yes	280	86.7
	No	43	13.3
Number of comorbidities, median (range)		2 (0–5)	
Type of comorbidities	Hypertension	168	52.0
	Diabetes	35	10.8
	Atrial fibrillation	96	29.7
	Coronary heart disease	91	28.2
	Heart failure	23	7.1
Type of oral anticoagulants	Warfarin	300	92.9
	Others^a^	23	7.1
Months of oral anticoagulants consumption	2∼≤3	16	5.0
	3–6	194	60.1
	>6	113	35.0
Tobacco use		19	5.9
Alcohol intake		17	5.3

MVR, mitral valve replacement; AVR, aortic valve replacement; DVR, double valve replacement; TVR, tricuspid valve replacement.

^a^ Other oral anticoagulants included clopidogrel, aspirin, and rivaroxaban, etc.

### 3.2 Adherence to oral anticoagulants, medication belief, and knowledge on oral anticoagulant therapy

Based on the total MMAS-8 score, 17.3% of participants had low adherence, 47.1% had medium adherence, and 35.6% reported high adherence to oral anticoagulants. Overall, participants were moderately adherent (mean = 6.93, SD = 1.31). The majority of the participants (n = 235, 72.8%) believed that the necessity of taking oral anticoagulants outweighed their concerns about oral anticoagulants (medication belief score > 0). The participants reported moderate beliefs about the necessity of oral anticoagulants (mean = 17.12, SD = 2.89) and moderate concerns about taking oral anticoagulants (mean = 13.76, SD = 3.27). The mean total score of oral anticoagulant therapy was 5.00 (SD = 2.46) out of 10 points. Table 2 summarizes the score of the adherence to oral anticoagulants, medication belief, and knowledge on oral anticoagulant therapy.

**TABLE 2:** Scores of main variables.

Variables	Mean (SD)	Observed range	Possible range
Adherence to oral anticoagulants	6.93 (1.31)	1.25 to 8	0 to 8
Medication belief (median)	4	−10 to 18	−20 to 20
Necessity	17.12 (2.89)	8 to 24	5 to 25
Concerns	13.76 (3.27)	5 to 22	5 to 25
Knowledge on oral anticoagulant therapy	5.00 (2.46)	0 to 10	0 to 10

SD, standard deviation.

### 3.3 Correlations between the main variables

Correlations between the main variables are shown in Table 3. Necessity belief (*r* = 0.655, *p* < 0.01) and knowledge of oral anticoagulant therapy (*r* = 0.459, *p* < 0.01) were positively associated with adherence to oral anticoagulants. Concern belief was significantly negatively correlated with adherence to oral anticoagulants (*r* = −0.588, *p* < 0.01).

**TABLE 3:** Correlation matrix of main variables.

Variables	1	2	3	4
1. Adherence to oral anticoagulants	1			
2. Necessity belief	0.655^a^	1		
3. Concerns belief	−0.588^a^	−0.486^a^	1	
4. Knowledge on oral anticoagulant therapy	0.459^a^	0.696^a^	−0.455^a^	1

^a^ *p* < 0.01.

### 3.4 Test of mediation effects

**FIGURE 2:**
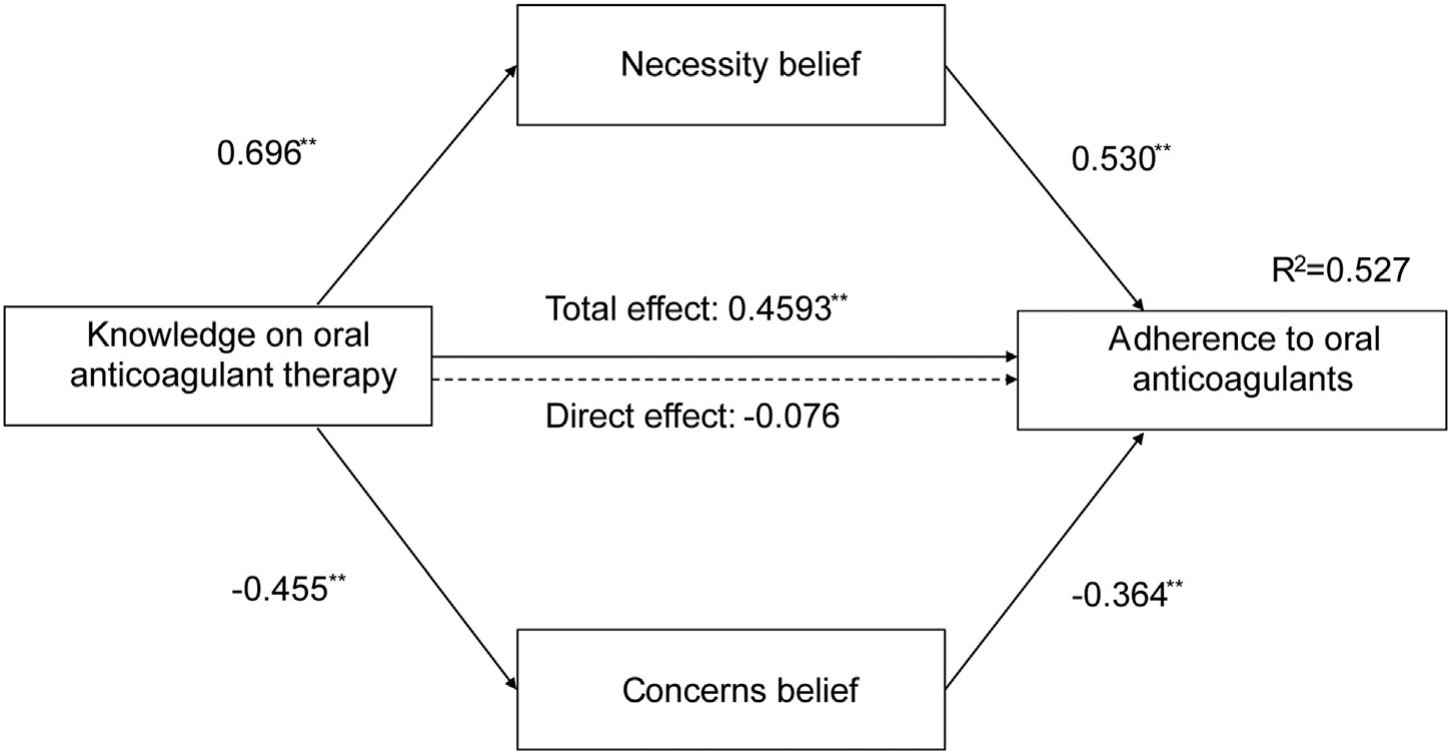
Final model and standardized model paths.

The mediation effects of medication belief on the relationship between knowledge of oral anticoagulant therapy and adherence to oral anticoagulants are presented in Table 4. The total effect of knowledge about oral anticoagulant therapy on adherence to oral anticoagulants was found to be significant (*B* = 0.244, *SE* = 0.026, *p* < 0.001). The total indirect effect of mediators (i.e., necessity belief and concerns belief) was statistically significant after controlling for the covariates of demographic and clinical characteristics (*B* = 0.284, *SE* = 0.032, 95% *CI* [0.224, 0.352]), while the direct effects of knowledge about oral anticoagulant therapy on adherence were not significant (*B* = −0.04, *SE* = 0.029, *p* = 0.167). Therefore, these results indicated a significant full mediation effect of medication belief on the relationship between knowledge about oral anticoagulant therapy and adherence to oral anticoagulants. The standardized path coefficients are shown in Figure 2. In sum, knowledge of oral anticoagulant therapy and medication beliefs jointly accounted for 52.7% of the variance in adherence to oral anticoagulants.

**TABLE 4:** Unstandardized total, direct and indirect effect estimates.

Variables	*B*	*SE*	*P*	95% confidence interval
Total effects				
Knowledge → adherence	0.244	0.026	<0.001	0.192, 0.296
Direct effect				
Knowledge → adherence	−0.04	0.029	0.167	0.017, −0.076
Total indirect				
Knowledge → adherence	0.284	0.032	-	0.224, 0.352
Specific indirect effect				
Knowledge → necessity belief→ adherence	0.196	0.027	-	0.147, 0.253
Knowledge → concerns belief→ adherence	0.088	0.015	-	0.061, 0.120

95% confidence intervals based on 5000 bootstrapping samples.

## 4 Discussion

The aim of this study was to investigate the prevalence of medication adherence, knowledge and medication belief and the mediating effect of medication belief on the relationship between knowledge and medication adherence among patients with BHV replacement. We found that 35.6% of patients with BHVs showed high adherence to oral anticoagulants, and 17.3% of patients had low adherence. Both patients’ knowledge and medication beliefs were positively associated with adherence to oral anticoagulants. Moreover, our results identified that medication belief mediated the relationship between knowledge and medication adherence. To the best of our knowledge, this is the first study designed to reveal the underlying mechanisms of medication adherence among patients with BHV replacement. This study can inform the design of interventions aimed at promoting patients’ adherence to oral anticoagulants after BHV replacement in the immediate postoperative period.

In this study, only one-third of our participants were highly adherent, indicating suboptimal medication adherence among patients with BHV replacement. Compared with previous studies conducted among patients taking oral anticoagulants (Ababneh et al., 2016; Smet et al., 2018; Wang et al., 2018), the rate of medication adherence in our study was relatively lower. In the Ababneh et al. (Ababneh et al., 2016) survey, approximately 54% of the participants were highly adherent to warfarin. Another study conducted among Chinese patients who underwent MHV replacement demonstrated that 86.8% of patients reported optimal adherence to anticoagulant treatment (Wang et al., 2018). This difference may be explained by the different methods of measurement and different sample characteristics. When comparing our results with prior studies, one should be taken into consideration that our sample underwent BHV replacement (i.e., short-term oral anticoagulant treatment). Patients with short-term medication treatment may have a limited frequency of visits. As a result, communication between patients and healthcare providers is limited in patients with BHV replacement, which may potentially lead to patients’ low medication adherence (Wu et al., 2019).

Our results showed that patients with BHV replacement had a medium level of knowledge about oral anticoagulants and further demonstrated a positive correlation between knowledge and medication adherence. This is in line with Smet et al. (2018) and Ayele et al. (2022), who found that knowledge was significantly associated with medication adherence. Patients with adequate knowledge about medication treatment were more likely to know the purpose of medication, the management of adverse events, interaction with other drugs and diet, and effect monitoring, which contributed to better medication adherence. In addition, based on the KAP model (Dong et al., 2022), mastering knowledge of oral anticoagulants is the first step toward performing medication adherence behavior. In our study, the knowledge related to oral anticoagulants was low compared with the study by Park and Jang (2021). This may be because knowledge about medication was associated with the medication intake period (Taylor et al., 2020); that is, patients’ knowledge increased over time (Taylor et al., 2020). Specifically, the longer the medication intake period, the better the patients’ knowledge. Most participants (65%) in our study took oral anticoagulants for a short period (<6 months), while Park and Jang (2021) study involved many patients (77.3%) who had taken warfarin for more than 3 years. Moreover, the majority of the participants in the current study were not highly educated; thus, they may not have enough cultural capital to master optimal medication knowledge (Chew et al., 2021). Our result was similar to the study by Cao et al. (2020), who found a moderate level of knowledge about warfarin therapy among patients with heart valve replacement regardless of MHVs or BHVs.

This study confirmed that medication belief was significantly associated with adherence. Specifically, necessity belief is positively linked to medication adherence, while concern belief has the opposite effect, which was consistent with other published studies reporting that a strong medication belief had a positive impact on medication adherence (Chua et al., 2018; Yoo et al., 2023). The overall score of medication belief was greater than “zero”, indicating that patients perceived more benefits than risks of oral anticoagulants. A possible explanation might be that the majority of patients in our study consumed anticoagulants for less than 6 months. Prior studies have indicated that patients with long-term anticoagulant therapy are more likely to experience medication-related adverse events due to a longer drug administration period (Park and Jang, 2021). Conversely, patients with BHV replacement receiving short-term anticoagulant therapy were less likely to suffer adverse events, which resulted in few concerns about medication.

As we expected, medication belief fully mediated the relationship between knowledge and adherence to oral anticoagulants. In other words, adequate medication knowledge helped to improve patients’ necessity belief and decrease their concerns belief, which in turn promoted medication adherence. This is supported by existing research findings (Hilliard et al., 2015; Qiao et al., 2020) showing that medication belief serves as a mediator to enhance patients’ medication adherence. Our results implied that the benefits of knowledge were translated to adherence through belief, highlighting the crucial role of medication belief. According to the KAP model (Kwol et al., 2020), attitude and belief are the nearest antecedent variables affecting behavior, as they are associated with one’s motivation. As such, only providing medication knowledge was not enough to efficiently enhance patients’ adherence, and healthcare professionals should make efforts to help patients appropriately understand the necessities and benefits of oral anticoagulants and address their concerns about potential adverse effects.

This study has several limitations. First, due to the nature of the cross-sectional design, we cannot infer any causal relationships between variables. Future research may be performed by using longitudinal or experimental designs to verify the findings in our study. Second, the generalizability of our results was limited, as the participants were recruited from only one hospital in China. Further research involving participants from other contexts, cultures, and countries is needed. Third, data were obtained from self-reported instruments, and patients were required to recall their medication adherence, which may lead to recall bias and social desirability bias.

## 5 Conclusion

This study found low adherence to oral anticoagulants among patients after BHV replacement. Moreover, medication belief can be one possible mediating mechanism underlying the link between knowledge and adherence to oral anticoagulants. Our study contributed to the limited research exploring medication adherence in patients with BHV replacement and established several predictors that help inform health professionals in designing interventions to improve patients’ medication adherence.

## Data Availability

The raw data supporting the conclusion of this article will be made available by the authors, without undue reservation.
